# Ex Vivo Systems to Study Chondrogenic Differentiation and Cartilage Integration

**DOI:** 10.3390/jfmk6010006

**Published:** 2021-01-05

**Authors:** Graziana Monaco, Alicia J. El Haj, Mauro Alini, Martin J. Stoddart

**Affiliations:** 1 AO Research Institute Davos, Clavadelerstrasse 8, CH-7270 Davos Platz, Switzerland; graziana.monaco@aofoundation.org (G.M.); mauro.alini@aofoundation.org (M.A.); 2 School of Pharmacy & Bioengineering Research, University of Keele, Keele ST5 5BG, UK; A.ElHaj@Bham.ac.uk; 3 Healthcare Technology Institute, Translational Medicine, School of Chemical Engineering, University of Birmingham, Birmingham B15 2TH, UK

**Keywords:** bioreactors, osteochondral, integration, tissue engineering

## Abstract

Articular cartilage injury and repair is an issue of growing importance. Although common, defects of articular cartilage present a unique clinical challenge due to its poor self-healing capacity, which is largely due to its avascular nature. There is a critical need to better study and understand cellular healing mechanisms to achieve more effective therapies for cartilage regeneration. This article aims to describe the key features of cartilage which is being modelled using tissue engineered cartilage constructs and ex vivo systems. These models have been used to investigate chondrogenic differentiation and to study the mechanisms of cartilage integration into the surrounding tissue. The review highlights the key regeneration principles of articular cartilage repair in healthy and diseased joints. Using co-culture models and novel bioreactor designs, the basis of regeneration is aligned with recent efforts for optimal therapeutic interventions.

## 1. Introduction

**Figure 1:**
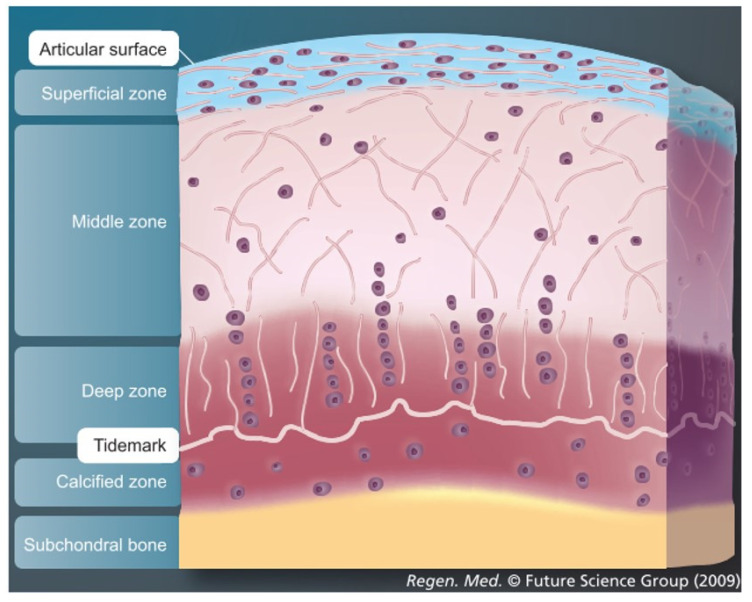
Zonal organization in physiological articular cartilage and subchondral bone showing a schematic distribution of cells and collagen fibril of superficial, middle and deep zones. Articular cartilage image modified with permission from M.J. Stoddart et al., 2009 [1].

Articular cartilage, which covers the osseous ends in diarthrodial joints, is an anisotropic tissue with a complex structure. Mature tissue is constructed of four layers: surface zone, middle zone, deep zone and calcified zone [1,2,3]. Each zone has a well-defined structural, functional and mechanical property that responds to different stimuli and is populated by a distinct cell phenotype that secretes different proteins and generates a well-defined organization of collagen fibers in each zone [4,5,6,7,8] (Figure 1). The main cell type resident in articular cartilage tissue is the chondrocyte and its main function is to maintain the extracellular matrix. The extracellular matrix in healthy cartilage consists predominantly of type II collagen fibers (>90%) with lesser amounts of type VI, IX and XI collagen [1]. In addition, to the collagenous molecules that provide a mesh-like framework responsible for the tensile properties, there are the non-collagenous molecules represented by a proteoglycan component. These confer the shock-absorbing properties of the matrix, due to the highly sulfated aggrecan monomers attached to the hyaluronic acid and to the link protein. The cartilage environment, therefore, is a hydrophilic environment that absorbs and retains large amounts of water. For this reason, 60–85% of cartilage tissue is made up of water. Moreover, as the weight bearing material of diarthrodial joints, the main function of articular cartilage is to produce a low friction surface capable of withstanding in vivo load in the mega Pascal range [9].

As articular cartilage is an avascular, aneural and alymphatic tissue, it is highly suited to dissipate and absorb load. However, the low metabolic activity of the mature tissue exerts a detrimental effect on its regeneration. Therefore, articular cartilage once damaged through trauma or disease has limited repair capacity.

### Cartilage Defects and Healing Response

Cartilage defects can be divided into three major classes: partial thickness defects, full thickness defects and osteochondral defects depending on the depth of the damage [10]. In partial or full thickness defects, the damage is restricted entirely to the cartilaginous tissue and does not penetrate the subchondral bone. Partial thickness defects differ from full thickness defects because they do not span the whole depth of the articular cartilage, while full thickness defects, although affecting the whole thickness of the articular cartilage, do not penetrate the subchondral bone [11]. Cartilage defects present a unique clinical challenge due to its poor self-healing capacity, largely dependent on its’ avascular nature that impede the blood cells and bone marrow MSCs from the surrounding environment reaching the defect and contributing to the healing response which normally occurs in vascularized tissue [12,13]. Moreover, cartilage tissue has a very low cell number content and the main cellular component, the chondrocyte, has a limited metabolic activity, proliferation and biosynthesis [14]. If cartilage damage is left untreated, not only the surrounding cartilage will be pathologically affected, but also the subchondral bone [15].

In osteochondral defects, the damage penetrates the subchondral bone and this event enables a rudimentary healing response. Blood first enters the lesion from damaged vasculature or from bone marrow and a fibrin clot is formed [13,16,17]. Platelets are trapped and release bioactive factors such as platelet derived growth factor (PDGF) and transforming growth factor beta (TGF-β). These factors then attract vessels and mesenchymal progenitors into the defect [17]. Unfortunately, this repair response often leads to the generation of a mechanically inferior fibrocartilage-like repair tissue, which is unable to withstand normal joint load and ultimately degenerates further [18]. Untreated defects, or failed treatments, can progress to osteoarthritis, which ultimately can lead to total joint replacement.

## 2. Therapeutic Interventions to Attempt Articular Cartilage Repair

**Figure 2:**
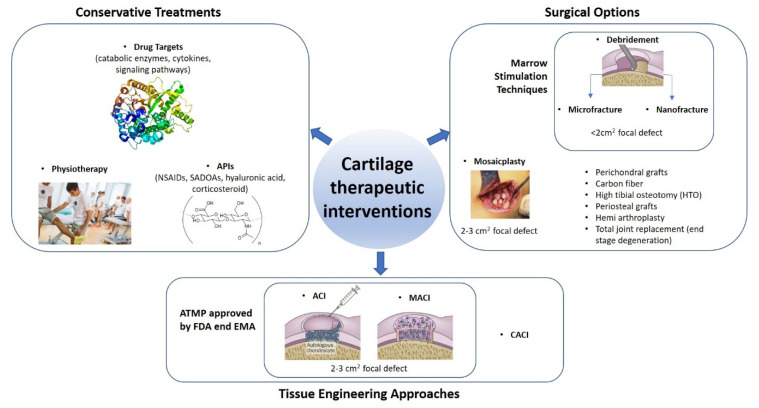
Therapeutic interventions to treat articular cartilage injuries of different size and severity. Defects with limited severity are treated by conservative treatments. Defects < 2 cm^2^ are treated with microfracture and nanofracture. Defects (2–3 cm^2^) are treated with mosaicplasty, autologous chondrocyte implantation or matrix-assisted autologous chondrocyte implantation (ACI/MACI). Single or multiple larger (2–4 cm^2^) defects are treated with grafts. End-stage osteoarthritic degeneration is treated by total joint replacement (TJR). Mosaicplasty photo is used under the terms of the Creative Commons Attribution-NonCommercial-No Derivatives License (CC BY NC ND). ACI, MACI and debridement pictures adapted by permission from Makris et al. 2015 [35]. Magnetic fields picture is reprinted with permission from Fini et al., 2005 [24]. Physiotherapy picture is licensed under the Creative Commons Attribution-Share Alike 4.0 International license.

Articular cartilage repair is an issue of growing importance. Despite the increasing number of therapeutic approaches, the number of total knee replacements will jump from 700,000 to 3.48 million annually by the year 2030 [19] and as revision surgery can be very complex and challenging, any intervention that can reduce or delay joint replacement will be of benefit. Trauma, osteoarthritis and osteochondritis are the most common causes of cartilage damage that lead to pain, swelling and impaired movement of the joint [20]. The choice of the therapeutic approach depends on the severity of the injury (Figure 2). Conservative treatments aim to reduce pain and improve joint mobility when the severity of the chondral lesion is limited [21,22,23,24,25,26]. In larger cartilage defects, depending on the nature, size and location of the lesion, more invasive surgical approaches are required [22,27]. Marrow stimulation techniques aim to introduce a source of reparative cells from the underlying bone (Figure 2). Microfracture is a modification of the Pridie drilling Technique [28], developed by Steadman et al. [29,30], that creates multiple holes in the exposed bone, which allows bone marrow cells to enter the defect and to differentiate into the desired tissue [30]. Microfracture shows a positive outcome seven years post-surgery [31]. Unfortunately, the de novo tissue is mainly composed of fibrous tissue with predominant type I collagen and limited type II collagen and lacks the organized structure of native articular cartilage. This leads to inferior mechanical capacity, poor integration and tidemark migration [32,33]. Newer developments, such as nanofracture, reduce some of these poor outcomes [34].

Mosaicplasty, first described in 1993 [36], fills the articular cartilage defect with osteochondral plugs but donor site morbidity is a concern and cartilage regions rarely show reasonable lateral integration [37,38,39,40]. High tibial osteotomy (HTO) [41], periosteal grafts [42,43,44,45,46] and total joint replacement are surgical solutions for severe damage [47]. 

Autologous Chondrocyte Implantation (ACI) involves the use of autologous chondrocytes isolated from a cartilage biopsy and secured in the defect using a periosteal flap [48]. Collagen-covered ACI (CACI), a second generation ACI, aimed to replace the periosteal flap with a bilayer type I/III collagen membrane [49]. Matrix-induced autologous chondrocyte implantation (MACI), a third more advanced scaffold-based approach, uses biodegradable collagen matrices seeded with the chondrocytes and anchored into the defect with fibrin glue. With this approach, there is a reduction in surgical complications as it is possible to operate by mini-arthrotomy [20,35,49,50]. In addition, chondrocytes cultured in a 3D environment, are less prone to dedifferentiation and therefore produce a more hyaline-like cartilage [51]. ACI and MACI have been approved by the Food and Drug Administration (FDA) and by the European Medicines Agency (EMA) as Advanced-Therapy Medicinal Products (ATMPs) [35,48,49] and has been adopted by NICE in the UK in November 2017 as a preferred treatment for OA lesions of a certain size. The donor site morbidity due to the required cartilage biopsy remains an issue, as does the small size of harvestable cartilage associated with a low chondrocyte density and their limited in-vitro expansion potential [49]. Therefore, a very small number of cells are available for ACI whereas a larger number may have more success in producing hyaline-like cartilage repair tissue. 

Mesenchymal stem/stromal cells (MSCs) also described as skeletal stem cells (SSCs), are a potential an alternative cell source for cartilage repair and are relatively easy to isolate from a variety of tissues. [52,53]. MSCs are considered to be immunologically inert and have immuno-modulating, proliferative and regenerative potential [54,55,56]. Therefore, MSCs are attractive candidates for cell therapy, with different MSC treatments currently progressing forward through to FDA approval for treatment of multiple conditions [57,58,59,60]. Based on the initial work of Friedenstein and Caplan, human bone marrow-derived MSCs (hBMSCs) are the best described and most advanced cells in clinical settings that can differentiate into cartilage or bone [61,62,63,64,65]. Human BMSCs, compared to chondrocytes, can be easily harvested from bone marrow aspirate with far less risk than mini-arthrotomy, which may lead to infection. They can then be easily expanded in monolayer culture with far less risk of undesired differentiation, unlike chondrocytes, which tend to adopt a fibroblast-like phenotype following expansion [66,67]. Indeed, it has been proposed that MSCs are capable of maintaining their ability to produce useful cartilage-like repair tissue longer than chondrocytes after monolayer expansion and also maintain a higher proliferation rate [68,69,70]. hBMSCs derived from patients with advanced osteoarthritis (OA) maintained a chondrocytic phenotype in a polyglycolic acid scaffold in the presence of TGF-β3, as assessed by ex vivo production of proteoglycan and type II collagen [71,72]. Another beneficial effect of MSCs, is their ability to selectively migrate to diseased tissues and organs and modulate inflammation [73,74,75]. Although how injected cells can be correctly targeted to the cartilage defect, as opposed to the synovium, is still a major challenge. In the case of cartilage repair, hBMSCs might enable a targeted repair system that promotes trophic effects through the release of synthetic, anti-inflammatory, proliferative and regenerative factors directly into chondral lesions [76]. By creating a regenerative environment with the release of chemotactic factors [77], BMSCs might also recruit endogenous stem cells to the cartilage defect and aid in the regeneration of damaged tissue [78]. Currently, autologous MSC therapies for cartilage repair are being investigated in combination with autologous chondrocytes as a co-therapy approach. Early trials are indicating improved cartilage regeneration even though the MSCs themselves do not appear to survive.

Despite the encouraging results and advancements in cartilage tissue engineering, much remains to be investigated, such as the feasibility of the clinical translation, the specific benefits of the different cell types (chondrocytes, stem cells, hBMSCs), the choice of the most suitable cell source, the ideal cells number to promote cartilage regeneration, the cost-effectiveness of the whole method, the long-term safety and efficacy. Therefore, there remains a critical need to develop more effective therapies for cartilage regeneration through the development of tissue engineering or regenerative medicine approaches. For this, to be successful, improved in vitro culture models will be required.

## 3. Tissue Engineering of Osteochondral Implants

**Figure 3:**
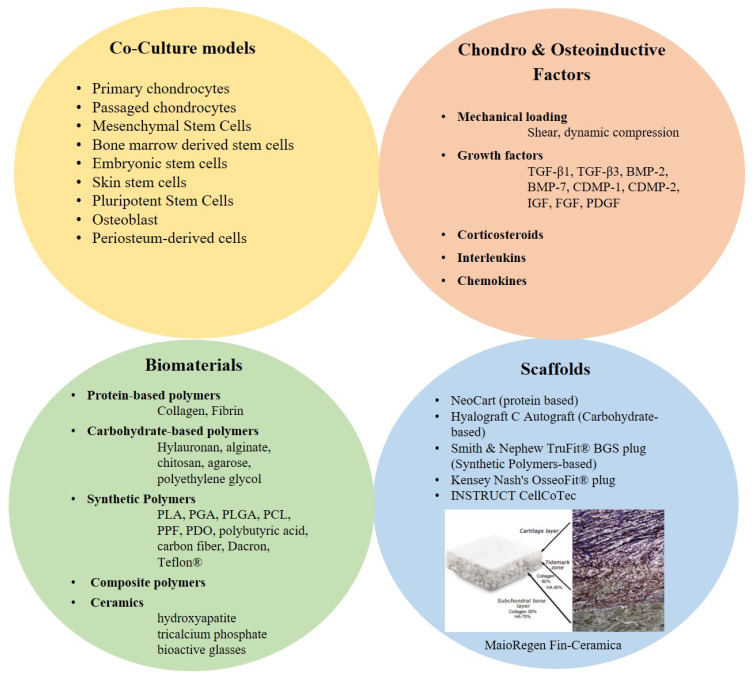
Tissue engineering solutions for osteochondral implants. Co-culture models, biomaterials, scaffolds and chondro/osteoinductive factors and signals are the most important parameters of tissue engineering approaches to osteochondral repair. Scaffold image MaioRegen, Fin-Ceramica modified with permission from E. Kon et al., 2010 [82].

One of the main problems of osteochondral lesions is the fibrocartilage formation that does not protect the subchondral bone from further degeneration [79]. Despite the available surgical options for osteochondral lesions there are still limitations in the regeneration and healing process [80,81]. As such, the future of treating osteochondral defects may lie in providing novel biologic solutions for cartilage and bone regeneration (Figure 3). 

To identify these regenerative approaches using biologic approaches, we need better models to test and screen new potential therapeutic approaches. Currently, these screening strategies rely on simple in vitro models which do not contain the full elements required for in vitro testing. To develop an osteochondral implant a multidisciplinary and multistep approach is required which includes; the selection of a proper cell type, such chondrocytes, mesenchymal stem cells or pluripotent stem cells; a suitable scaffold material, which could be protein-based material, carbohydrates-based material, synthetic-based material or composite polymers; a suitable technique that allows the production of scaffold structures with tissue specific mechanical properties, stiffness and porosity; chondro-inductive and osteo-inductive factors to enhance the cell differentiation and tissue formation; and finally, bioreactors to improve the nutrient delivery and provide relevant mechanical stimulation to the osteochondral tissue-engineered construct. 

The complex multi-disciplinary approach currently being developed to produce osteochondral substitutes is the most promising route to bring cartilage and bone regeneration from bench side to the clinics.

### 3.1. Importance of Reproducing the Zonal Organization of Articular Cartilage and Subchondral Bone

Articular cartilage in the human knee joint is organized in different zones: superficial zone, middle zone, deep zone and calcified zone [83]. The latter includes a layer also known as the tidemark which consists of a thin layer of mineralized tissue. Immediately below the calcified zone of articular cartilage, there is the subchondral bone plate and the subchondral trabecular bone, a vascularized tissue that contains the bone marrow cavity [84]. The zonal organization of articular cartilage and subchondral bone play an important role for osteochondral structure, function and responsiveness to different mechanical stimuli. During the transition from the superficial zone to the deep zone, the extracellular matrix is characterized by increased stiffness, proteoglycan content and hypertrophic chondrocytes [83]. The subchondral region shows a variable anatomy which differs in thickness, density and composition [84]. Most tissue-engineering strategies aim to regenerate osteochondral tissues by increasing the complexity of the biochemical and mechanical cues to approximate the native structure and collagen fibril orientation of the different zones of articular cartilage as well as the subchondral bone [85,86,87,88,89]. The improvement in the mimicking of the macro and microstructure of the osteochondral tissue will be one of the main goals for future osteochondral tissue engineering. Only in this way, it will be possible to reproduce a more structurally sound native tissue able to withstand the natural in vivo mechanical loading patterns. In addition, when stem cells derived from different sources are used, an important issue that still needs to be solved is the potential to differentiate into a hypertrophic phenotype [90,91,92]. As such, additional studies are needed to understand how to obtain stable articular chondrocytes from hMSCs.

### 3.2. Biomaterials & Scaffolds

One of the main challenges of osteochondral tissue engineering is to reproduce through biomimetic scaffolds, the complex architectures of two tissues that despite being physically interacting, have completely different properties [93,94,95]. Additionally, the interface between the two tissues, despite their mismatching mechanical properties, needs to remain intact during load. To mimic bone, it is necessary to use a mechanically stiff biomaterial that allows the production of bone matrix rich in type I collagen and hydroxyapatite (HA). In addition, a certain level of medium perfusion is required to mimic the in vivo vascularization and adequately support cell expansion. Pre-vascularization may even be a prerequisite. By contrast, native hyaline cartilage matrix consists of an avascular highly hydrated proteoglycan hydrogel rich of type II collagen network.

Different types of biomaterials and scaffolds have been developed to establish a three-dimensional structure that retains the seeded cells and provides mechanical support to guide the development of cartilage and bone over time. The biomaterials, in addition to satisfying the different mechanical requirements intended for cartilage or bone regeneration, need to be cytocompatible, biocompatible, biodegradable, noncytotoxic, mechanically responsive similarly to the native tissue, able to modulate cell proliferation and differentiation, functionalizable with an appropriate surface chemistry, shapeable into different sizes and forms and crosslinkable to modulate stiffness and biodegradation [96]. In addition, biomaterials intended for osteochondral tissue engineering, need to be chondro and osteo-conductive to be able to regenerate respectively hyaline cartilage and subchondral bone. 

The most successful approach currently used to mimic the osteochondral defects is an osteochondral construct with stratified multi tissue regions that can reproduce the zonal localization and organization of the native tissue. In this regard, different solutions have been proposed. A poly vinyl alcohol/gelatin-nano-hydroxyapatite/polyamide6 (PVA-n-HA/PA6) bilayer scaffold seeded with bone marrow stem cells implanted in rabbit, showed neocartilage formation in the PVA layer and reconstitution of the subchondral bone in the n-HA/PA6 layer [97]. A biphasic scaffold which consisted of hyaluronic acid and atelocollagen for the chondral phase and HA and beta tricalcium phosphate (β-TCP) for the bony phase has proved to be effective for repairing osteochondral defects, when implanted in the knee joint of a porcine model [98]. MaioRegen, a 3D biomimetic scaffold produced by Fin-Ceramica S.p.A. in Italy, was created by nucleating type I collagen fibrils with HA nanoparticles, in two configurations, bi- and tri-layered, to reproduce, respectively, chondral and osteochondral structure [99]. This scaffold, tested in chondral and osteochondral defects in horse and sheep, promoted the growth of trabecular bone and fibrocartilaginous tissue and with good integration [82,100]. The same scaffold has been also used in a clinical trial to heal large degenerative chondral lesions with an average size of 2.8 cm^2^ and showed the formation of subchondral bone and the cartilage repair tissue [101]. 

An interesting example of an osteochondral scaffold with a complex multilayer structure was developed by Lien et al. [102]. This scaffold design has been shown to be reasonable for bone and articular cartilage repair. The scaffold structure consisted of four layers: a porous ceramic layer to mimic the bony zone, a dense ceramic layer to prevent blood vessel penetration and to resist shear stresses, a porous ceramic layer to fix bone with cartilage and a porous gelatin layer as the cartilage facing component. The prevention of blood vessel penetration into the cartilage layer from the bony layer was important to prevent the ingrowth of blood vessels and the growth of the bone in the cartilage layer [103,104].

Among different products already in use for cartilage tissue engineering, INSTRUCT (*CellCoTec, Bilthoven, Netherlands*) a poly(ethylene oxide- terephtalate)/poly(butylene terephtalate) (PEOT/PBT) scaffold seeded with primary autologous chondrocytes and bone marrow cells has been shown to be promising for cartilage regeneration [105]. PEOT/PBT based scaffold was designed to be mechanically functional during compressive dynamic loading of 10 MPa, mechanically matching articular cartilage. The innovative one surgery cartilage repair approach allows to conduct the cell seeding procedure at the point-of-care by taking a patient’s cartilage biopsy plus bone marrow aspirate. Both cells sources are processed, isolated, mixed, resuspended in fibronectin and seeded into the PEOT/PBT scaffold by a semi-automated machine, the INSTRUCT cell processor. 

### 3.3. Chondro-Inductive and Osteo-Inductive Factor/Molecules/Signals

Different chemical cues have been used to induce and promote osteochondral tissue formation. Physiological stimuli are mainly used to stimulate stem cells, immature bone or cartilage cells to differentiate, grow, mature and form healthy tissue. Among the growth factors, members of the TGF-β superfamily are often used to stimulate cartilage repair. TGF-β1, TGF-β3, bone morphogenetic protein (BMP)-2, BMP-7 and cartilage-derived morphogenetic proteins (CDMP-1 and CDMP-2) have been used to induce chondrogenic differentiation in MSCs and stimulate production of cartilage extracellular matrix [106,107,108]. Most likely the effect of these TGFβ family and BMP growth factors on the transcription factor Sox9 may be the key in controlling chondrogenesis. In fact, when MSCs were manipulated to overexpress Sox9, an increased proteoglycan and type II collagen deposition, as well as prevention of terminal differentiation, with an overall enhancement of the chondrogenesis was observed [109]. In addition, much interest has centered on a group of proteins called Bone Morphogenetic Proteins. In particular, BMP-2 has been used in orthopedic applications, mainly for stimulating bone growth, either in the setting of fracture healing or spinal fusion. Additionally, BMP-7 has been shown to stimulate cartilage matrix synthesis, acting synergistically with other anabolic growth factors, and also inhibits catabolic factors, such as matrix metalloproteinase-1 (MMP-1), MMP-13, IL-1, Il-6, and IL-8 [110,111]. Insulin-like growth factor (IGF) and fibroblast growth factor (FGF) are used for articular cartilage tissue engineering [106,107,108,112,113]. IGF-1 helps to maintain articular cartilage integrity and induces anabolic effects while decreasing catabolic ones. IGF-1 works better in combination with other growth factors, such as TGF-β and BMP-7 [114]. Fibroblast Growth Factor 2 is an intrinsic chondroprotective agent that suppresses ADAMTS-5 and delays cartilage degradation [112]. Platelet derived growth factor (PDGF) is a chemotactic factor for mesenchymal cells and has been shown to promote the formation of cartilage, to increase proteoglycan production and cell proliferation and to suppress IL-1β by downregulating NF-κB signaling [114]. Corticosteroids [115,116], and interleukins (IL) [117,118,119] has been found to promote extracellular matrix (ECM) synthesis. Some of the already mentioned chemical cues have been used to replace the effect of the paracrine factors released by chondrocytes in co-culture with MSCs.

The dose and spatial-temporal release of the growth factor could have a significant effect on therapeutic efficacy. As such, the development of a suitable method which allows accurate control of the released concentration and the specific location of the growth factor in the de novo tissue, is crucial to achieve clinical success. Due to the short half-life of many growth factor administered by local injection [120,121], other delivery techniques that would grant a better and more controlled release (longer half-life at the suitable concentration) should be developed. Zonal dependent controlled delivery of growth factors by the scaffold is crucial for engineering composite tissue structures, such as osteochondral constructs [122]. Microspheres could represent a strategy to achieve a better spatial control of growth factor delivery in 3D scaffolds [122]. It was observed that PLGA microspheres-based scaffold loaded with opposite gradient of BMP-2 and TGF-β1 and seeded with stem cells, demonstrated good osteochondral tissue regeneration [123]. An alternative to growth factor delivery, is localized gene-therapy that allows the delivery of a gene encoding the growth factor needed in a temporal controlled way [113,124,125,126,127].

Finally, the guided delivery of growth factors, drug and even cells within scaffolds can be achieved also by using superparamagnetic nanoparticles [128,129,130,131,132]. 

## 4. Pellet Culture: A Simple Cartilage Model

A current standard, widespread and simple model to induce in-vitro chondrogenic differentiation of bone-marrow derived MSCs, is the pellet culture described by Johnstone et al., 1998. This culture model has been shown to be effective in achieving chondrogenic differentiation of MSCs by providing a 3D environment that allows a close contact among the cells similar to those that occur in precartilage condensation during embryonic development [133]. However, the close cell-cell interaction allowed by this model, is not sufficient to obtain a stable articular chondrogenesis differentiation of MSC. In addition to cell-cell interactions, a defined medium supplemented with bioactive factors that drive chondrogenesis, such as TGF-β1 and dexamethasone, is needed [134]. However, a limitation of this cartilage model, is that TGF-β induced-chondrogenesis over time leads to hypertrophy of MSCs, similarly to that observed during bone formation via endochondral ossification [135,136]. Hypertrophy (increase in size) is the final stage of the terminal differentiation of chondrocytes during endochondral ossification and allows for the conversion of the cartilage tissue into bone. The size of the cells during the hypertrophic process can increase up to twenty times [137,138]. Chondrogenic markers type II collagen and aggrecan are down-regulated [137,139]. Hypertrophic and osteogenic markers type I and type X collagen, matrix metalloproteinase 13 (MMP13), runt-related transcription factor 2 (Runx2) [137], and alkaline phosphatase (ALP) start to be upregulated [135]. In a later stage vascular endothelial growth factor (VEGF) is expresses, which causes invasion of blood vessels [137] and calcification of the cartilaginous tissue. Chondrocytes grown in high density pellet cultures show a different behavior compared to MSCs. First, they maintain their phenotype without progressing towards hypertrophy. In addition, they are more effective than MSCs in producing cartilage-like tissue which is mechanically superior and contains higher levels of aggrecan and type II collagen [140,141]. Organoid tissue derived from iPS cells are also proving useful tools in osteoarthritis research [142]. Microtissues are being used as a further development of the pellet model to reduce the necrosis issue [143]. Furthermore, a 3D microPellet culture model in a high-throughput in-well configuration has been used as a screening system to facilitate the in vitro selection of pro-chondrogenic treatments [144]. While useful for high throughput screening, the pellet culture presents disadvantages. Cells can potentially dedifferentiate or necrotize in the central region of the pellet and chondrogenic differentiation is not evenly distributed [143,145,146,147]. However, to better approximate the structure of the native hyaline cartilage and to more similarly reproduce the tissue functionality, more complex culture models are needed. 

## 5. Cartilage Explant Culture and Cartilage Integration

**Figure 4:**
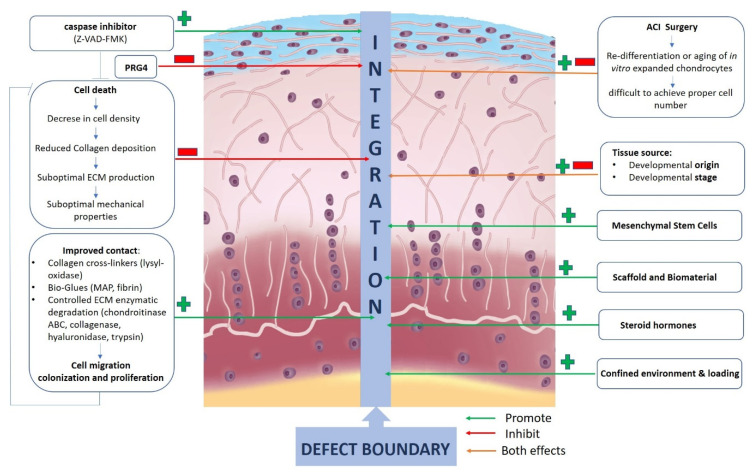
Factors involved in cartilage integrations. Articular cartilage image modified permission from M.J. Stoddart et al., 2009 [1].

One critical aspect of cartilage regeneration is the poor integration of the newly formed tissue with the native surrounding articular cartilage that leads to poor or failed tissue repair [148,149,150]. Several factors are involved the lack of cartilage integration (Figure 4). First, the partial cell death that occurs at the defect boundary and at the edge of the graft reduces the physiological cell density; this leads to a sub-optimal matrix production and collagen biosynthesis with inferior mechanical properties [89,151,152]. Indeed, it has been demonstrated that decellularized cartilage repopulated by physiological numbers of chondrocytes recovered integration function which provides evidence that the integration is due to the presence of physiological cell density [153,154,155]. 

**Figure 5:**
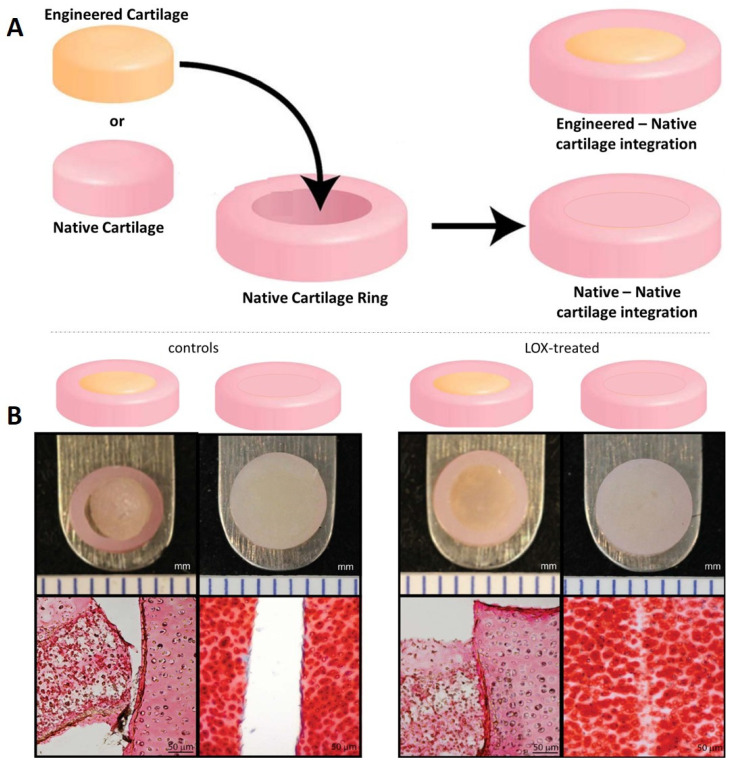
Schematic representation of the approaches examining integration of native or tissue engineered cartilage to native cartilage (**A**) and positive effect of lysyl oxidase (LOX) as enhancer of cartilage integration between native—native and tissue engineered—native cartilage. Native articular cartilage was harvested from distal femurs of one-week old male calves and tissue rings were created (**B**). Cartilage ring was filled with native tissue previously collected during the ring preparation. In parallel engineered cartilage consisting of agarose gel seeded with chondrocytes was also used to fill the cartilage ring. The tissue integration of native to native and engineered—native cartilage was evaluated in presence and absence of lysyl oxidase which induce collagen cross-linking across cartilage interfaces. Images modified from Athens et al., 2013 [160] under the terms of the Creative Commons Attribution License.

To perform in vitro studies on integration, cartilage explant culture is often used (Figure 5). Cartilage explant can be prepared in different ways. Osteochondral disks can be harvested by mosaicplasty technique and chondral disks can be obtained by removing the subchondral bone with a scalpel [156]. Alternatively, articular cartilage explants can be directly harvested from the metacarpo-phalangeal joints of calves and full thickness cartilage explants of 8 mm diameter can be prepared using a dermal biopsy punch and scalpel [157]. Then, full or partial depth circular holes can be cut by using a biopsy punch to form annuli of tissue [158]. The cartilage explants and the cartilage defects can be kept under confined or unconfined environment under loading or static conditions. Yodmuang et al. observed that a minimum level of scaffold-cartilage integration is needed prior to the commencement of loading, although, the exact threshold is still unknown [159]. The strength of the tissue integration can be biomechanically evaluated through push-out tests, [157]. The cartilage explant culture, compared with pellet culture, offers a more complex and reliable environment to investigate cartilage regeneration and integration processes. Furthermore, it is more suitable for evaluating new therapies. Cartilage explants maintain the native organized structure of the superficial and middle zone and better preserve the biological cues, which allows cartilage repair with chondrocytes or with MSCs to be investigated and different biomaterials/hydrogels to be tested.

Cartilage integration can be improved by directly inhibiting chondrocyte death by using a caspase inhibitor, such as Z-VAD-FMK [161]. Unsuccessful re-differentiation or in vitro-aging of expanded chondrocytes collected from a patient biopsy during ACI surgery might also negatively affect the integration process [89,162,163,164,165,166]. As outlined above, MSCs, unlike chondrocytes, appear to be a promising cell source for cartilage repair and when transplanted in the osteochondral defect, they differentiate according to the environment making an important contribution to initial cartilage formation [167,168]. 

ECM composition and function can play an important role in integration and adhesion strength of repair. Some components of the synovial fluid may have a negative effect on integrative cartilage repair. An example is Proteoglycan 4 (PRG4), a glycoprotein synthesized within the superficial zone of articular cartilage to allow frictionless movement of opposing cartilage joint surfaces, which has been shown to inhibit cartilage integration. [169,170]. However, hyaluronic acid, another important component of synovial fluid with analogue lubricating function as PRG4, doesn’t inhibit the integrative process [171]. To facilitate the fusion process, a good contact at the defect boundary is of a great importance to assure healing and integration. To improve such contact, collagen cross-linkers such as lysyl-oxidase (Figure 4) [148,160], an enzymatic solution that act like a biological glue [171] or protein-based adhesive are available, including clinically approved fibrin gel [155,172,173]. Within the ECM component, important players of the integration process are collagen fibrils and the complex network they form. 

The proper balance between synthesis, deposition, processing and degradation of collagen macromolecules is critical for integrative cartilage repair. Indeed, the integration potential of vital and devitalized cartilage is directly correlated to the level of collagen deposition. Viable chondrocytes secrete matrix molecules that build the collagen network and a continuous deposition of these molecules at the defect boundary enhances the functional integration [174]. Steroid hormones such as testosterone increases in a dose dependent manner the level of integration and this mechanism could be related to the anabolic response of collagen turnover [175]. Controlled enzymatic degradation at the defect boundary by using collagenase, hyaluronidase, trypsin or chondroitinase ABC, is an alternative approach to improve cartilage integration [157,176,177,178,179]. Chondroitinase ABC ameliorate the initial healing response of articular cartilage by inducing an early transient increase in the local population of repair cells at the defect surface due to the facilitated cell migration through the enzymatically degraded proteoglycan matrix, followed by enhanced cell colonization, proliferation and ECM deposition [89,176,177,179,180,181,182,183,184]. 

Different strategies have been developed to successfully achieve vertical integration and fusion with the subchondral bone. Unfortunately, lateral cartilage-cartilage integration remains one of the most complex issue in cartilage repair that needs to be further studied to achieve successful and long-term integration.

## 6. Co-Culture Models

For osteochondral tissue engineering purposes, it is crucial to mimic as closely as possible the native tissue in terms of the natural cellular composition, distribution and beneficial biomimetic environmental conditions necessary for cell survival, proliferation and stable differentiation in cartilage or bone. As such, the cross talk and the organization of the cells within the tissue is essential for the tissue’s normal development, homeostasis and repair. The ability to manage and reproduce the complex architecture of the osteochondral tissue of the knee joint from a cell point of view is still challenging but represents one of the key factors for a successful tissue regeneration. Since most tissues in the body, including osteochondral tissue, consist of more than one cell type, the development of a suitable co-culture system becomes an important requirement to finally achieve a functional osteochondral implant both for clinical needs and for research purposes. Co-culture has proved to be a powerful in vitro tool to study the cellular interactions during normal physiology, homeostasis, repair and regeneration. As such, the co-culture system provides a precious opportunity to study, manage and finally exploit the cell-cell communications to understand how they influence the tissue formation, development and maintenance.

In cartilage tissue engineering, co-cultures generally consist of primary chondrocytes mixed with a less differentiated cell type, such as a passaged chondrocytes or stem cells [185]. The role of the primary chondrocytes is to induce the less differentiated cell type toward a more complete chondrogenic differentiation, without the addition of exogenous biomimetic stimuli applied to the undifferentiated cells alone [186]. Similar observations have also been seen during co-culture of MSCs and osteoblasts [187]. On the other end, the less differentiated cells, provide the high cellularity needed for new tissue formation and potentially secrete factors that enhance chondrocyte function. Promising results in generating hyaline de novo tissue has been observed in co-cultures of primary chondrocytes with passaged chondrocytes, embryonic stem cells, bone marrow derived stem cells or skin stem cells [185,186]. Periosteum represents another source of autologous stem cells, but the harvest is invasive and moreover yields a paucity of cells [188].

An in vitro co-culture of primary and expanded chondrocytes 1:4, seeded in poly(ethylene oxide- terephtalate)/poly(butylene terephtalate) (PEOT/PBT) based scaffold, was shown to be promising for neocartilage formation as, after 4 weeks of co-culture, neocartilage completely filled the pore spaces of the scaffold [189].

Two different studies have shown that co-cultures of primary chondrocytes and mesenchymal stem cells (MSCs) produced pellets with similar or higher matrix content than those formed by only primary chondrocytes [190,191]. This confirms the hypothesis that MSCs actively help cartilage formation by increasing the chondrocyte population through direct MSC differentiation. In addition, MSCs help the existing chondrocytes in phenotype maintenance [76,192]. In contrast with previous findings, it has been observed that MSCs during chondrogenic co-culture, most likely undergo apoptosis in place of chondrogenic differentiation [193]. As such, it has been hypothesized that MSCs will stimulate chondrocyte proliferation and maturation, but don’t actively contribute to cartilage formation [76,194,195]. In addition, in a non-contact co-culture system, it was also observed that MSCs can even downregulate chondrocyte differentiation [196]. 

Co-culture of cartilage explants with synoviocytes has been proposed as a mechanism by which additional inflammatory aspects can be investigated [197].

## 7. Microfluidics 

Microfluidic systems are gaining in interest due to the small fluid volumes and low cell numbers required [198,199]. Microfluidic encapsulation has also been proposed as a mechanism by which cells can be delivered to cartilage defects [200]. Microfluidic tools have been using to investigate inflammation models [201], to provide mechanical stimulation [202,203,204], and even to select MSCs with better chondrogenic potential [205]. These systems allow for high throughput analysis of multiple conditions. While offering several advantages, output measures can be limited, and improvements would dramatically increase the usefulness of these technologies. 

## 8. Osteochondral Explant, Osteochondral Defect and Culture Models

The first-described osteochondral models were not intended for in vitro use and were directly implanted in vivo [206,207]. Among the studies based on the osteochondral defects, rabbit is a commonly used animal model, but it present some disadvantages, as its cartilage thickness is approximately 0.3 mm thick [208] and its joint scale is significantly smaller than humans [209]. In addition, osteochondral lesions in smaller animals have the tendency to heal quickly if compared with larger animals [210]. As a result, the effectiveness of the treatment provided in small animal models may be attenuated or disappeared in large animal models. Thus, the outcome of the therapy will be difficult to interpret with a clear conclusion from small animal studies. [211]. To perform a reliable osteochondral tissue-engineering study, it is important to use tissue from larger animals that show cartilage thickness similar to humans [212]. The use of chondral graft in place of osteochondral grafts, such as fetal allografts [213] and adult costal ones [214], did not attract much attention, either experimentally or clinically [18] mainly for two reasons. First, there exist few sources from which this tissue can be obtained, and second, it is difficult to properly preserve chondral tissue transplants within a defect, which expose them to a high probability of loss. For these reasons this approach is not the preferred one [18].

Therefore to reduce the costs associated with larger sample size with anatomical tissue scales and to fully mimic the full depth of the repair environment, the development of an osteochondral ex-vivo culture model by producing partial or full defect (from animal or human tissues) would be of great value.. A well designed ex-vivo/in-vitro culture model has the advantage to better mimic the physiological in vivo environment without the need to use animals. Therefore, the relevance and the benefit of this approach in the current climate of animal welfare and 3R principles is clear.

In fact, the “3Rs” Principle, Replacement, Reduction and Refinement, is considered as the key strategy of a systematic framework aimed at achieving reductions in animal numbers used in regulatory and research in vivo studies. This principle is being increasingly incorporated into legislations, guidelines and practice of animal experiments in order to safeguard animal welfare [215]. Russell and Burch saw replacement as the ultimate goal for laboratory animal-based research, education and testing, with the other two, reduction and refinement, being more readily achievable in the short term [216]. Törnqvist et. al. state that the new in silico-, in vitro- and in vivo-methods all hold the potential for applying the reduction R and should be consequently coordinated at a strategic level [215]. As such, the newly developed ex vivo/in vitro models would be key in facing the problem of animal use for experimental purposes. 

There are key requirements for an osteochondral model, e.g., the accurate description of the type of the defect and the depth of the defect associated with a suitable characterization of the model and mimicking the physiological environment such as oxygen diffusion characteristics. Osteochondral models in the literature often describe only one type of defect [156], do not have a control over the defect depth, do not describe the depth of the defects [217], or they have limited in vitro model characterization [206,207].

**Figure 6:**
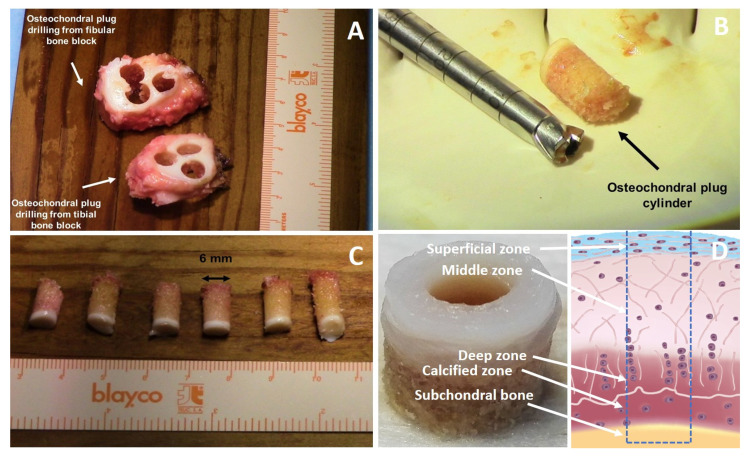
Autologous articular cartilage. Grafted from the upper tibiofibular joint and drilled (**A**) to create osteochondral plug cylinders 6 mm diameter and different thickness (**B**,**C**). Osteochondral defect of controlled depth is then obtained by drilling the middle region of osteochondral plug (**D**). Images modified from Espregueira-Mendes et al., 2017 [219] under the terms of the Creative Commons Attribution-NonCommercial-No Derivatives License (CC BY NC ND).

Control over defect depth is a critical requirement for the development of a reliable osteochondral culture model since the depth of the defect is crucial for the healing process as it is related to the qualitative and quantitative level of integration of the newly formed repair tissue into the surrounding environment [218]. The control over the depth of osteochondral explants and derived defects [219,220] is of a key importance as the subchondral bone plays an important role in the repair mechanisms (Figure 6). Subchondral bone and cartilage are closely related anatomically but also influence each other in the disease process [221,222,223,224,225,226]. Subchondral bone has also been identified as critical success factor for the microfracture procedure due to its primary involvement in the formation of repair/new tissue after cartilage repair treatments [227,228]. This topic has been addressed with different approaches. In the study carried out by Melle et al. 2011, the effect of subchondral bone on the healing process was evaluated during culture time and characterized by means of TRAP staining, calcein labeling and ALP activity measurements. They demonstrated that the osteochondral biopsies after 28 days of culture were different when compared to day 0 and concluded that the subchondral bone remained active during the culture. They also found that the osteochondral biopsy provides a more representative culture system to the native physiologic environment than the chondral only explants due to the presence of the subchondral bone that promotes a different expression pattern of cartilage-related genes, with particularly high type II collagen gene expression. This finding supports the hypothesis that subchondral bone has a crucial role during the healing process, as previously demonstrated [61,220,229,230]. 

Another important aspect in the development of an ex-vivo explant model is related to the size of the wound surface compared to the size of the explant. The lower this ratio is, the better the chondrocyte survival and the functionality of the whole explant will be. Cartilage that is explanted or otherwise damaged shows chondrocyte death at the wound edges [89,231,232]. As such, by minimizing the extension of the damaged area there is a greater chance to have a well-functioning tissue. The new culture models may be used to study the integration of the implant into the native surrounding environment represented by the defect or to screen different cell sources, biomaterials or tissue engineered constructs for their integration capability. 

During the development of an osteochondral culture models, another point necessary to consider is the different levels of oxygen and nutrients to which chondrocytes are exposed depending to the depth of the layer where the chondrocyte reside and the distance from synovial fluid. It is known that chondrocytes in vivo are exposed to a gradient of oxygen and nutrient supply [233]. For this reason, one advantage that makes the osteochondral culture model more similar to the in vivo environment compared to the conventional cartilage-only explant cultures is its capability to better reproduce the diffusion properties This means that the deep cartilage zone in the osteochondral explant is less exposed to oxygen and nutrients than in cartilage only explant as the molecules need to diffuse through the superficial and middle zone of the cartilage to reach the deep-zone [2,83]. This leads to a useful physioxia condition of the deep cartilage zone in osteochondral explant that better mimics the in vivo situation [233]. In fact, literature has already described the pivotal role of low oxygen as well as the related hypoxia-inducible factor-1(HIF-1a), which is a critical transcription factor, in chondrocyte survival, energy generation and matrix synthesis by articular and growth-plate chondrocytes during cartilage homeostasis [233]. Therefore, low oxygen tensions in the deep cartilage zone is an important factor required to modulate articular chondrocyte behavior in osteochondral explants.

Two other factors which support the development of an osteochondral model are that A good culture system should be able to keep the expression of hypertrophic markers low as in the osteochondral model proposed by Melle et al. 2012 [135,139]. 

Another advantage of the osteochondral transplants is their capability to survive for longer periods (some years), even after freezing or lyophilization [234,235,236,237,238,239]. The potential to use human tissue allows for a more clinically relevant representation [240].

With an increasing number of concepts emerging in the osteochondral tissue engineering disciplines, it will be necessary to better understand the molecular mechanisms behind the healing process by developing reliable high-throughput and cost-effective ex vivo models using human cells. To our knowledge, the osteochondral culture models so far described do not completely reproduce the classical gene expression level (type II collagen) and the biochemical composition (GAG level) of the native healthy articular cartilage and need further improvement.

## 9. Bioreactor Systems/Loading Devices Used for Osteochondral Applications

Articular cartilage is designed to withstand significant complex load and deformation during locomotion and other physical activities in vivo by providing a smooth, lubricated surface for articulation. Articular cartilage exhibits unique mechanical properties enabling it to transmit load to subchondral bone while providing the joint with a nearly frictionless articulation, thus protecting it from potential mechanical wear and damage [83]. Articular motion is an important aspect of mechanotransduction in synovial joints. The mechanical behavior of this tissue depends on the interaction of its fluid and solid components. Two major load-bearing macromolecules are present in the extracellular matrix of articular cartilage: type II collagen, a fibrillary molecule that confers resistance to tension, and proteoglycans, notably aggrecan. The interaction between the highly negatively charged cartilage proteoglycans and type II collagen provides the compressive and tensile strength of the tissue [83,241].

During mechanical loading of the joint, the rapid application of articular contact forces is the first step of motion, inducing an immediate local increase in interstitial fluid pressure, which causes the fluid to flow out of the ECM, generating a large frictional drag on the matrix [242,243,244,245,246]. In the second step of the motion, the compressive load is removed and the interstitial fluid flows back into the tissue. Due to the low permeability and high negative charges of articular cartilage, the fluids are retained in the ECM instead of being quickly squeezed out [245,247]. Joint motion and load are important to maintain normal articular cartilage structure and function. Inactivity of the joint has been shown to lead to the degeneration of cartilage [248] and this is relevant when considering standard static cell culture. 

Current culture models to investigate cartilage repair therapies are often highly simplified and critical in vivo signals such as load, are lacking. This limits the efficacy of in vitro tests, placing a higher burden on in vivo models. The importance of load and mechano-stimulation on musculoskeletal tissues and cells has long been recognized [249]. It is necessary to consider that mechanical loading is important not only during the development of the musculoskeletal system but also after development and is essential for the maintenance of healthy articular cartilage [250,251,252]. Physiological loads have been related to ECM production and affect the synthetic activity chondrocytes in vitro [253,254], sub-physiological loads have been shown to cause translational arrest [254,255]. Thus, mechanical stimuli are important for cartilage repair [256,257]. Cartilage constructs can also be mechanically stimulated in vitro to enhance chondrocyte matrix synthesis and remodeling [253,258], and to recapitulate zonal characteristics within the construct [259,260]. 

Integration of kinematic load into ex vivo osteochondral culture models would allow a closer representation of the in vivo environment [220]. Using an iterative approach, it would be possible to improve the developing culture systems by making them more similar to the real in vivo situation. This strategy could provide a new model to study healing or the regenerative processes of articular cartilage in a more joint-like environment, especially when various mechanical loading patterns can be applied to the model.

In recent years, there has been a considerable effort to produce bioreactors and loading devices [261,262,263,264,265,266,267]. The bioreactors might be a supporting tool to expose cells seeded in a scaffold structure or the whole tissues present in an ex-vivo explants, to different forms of mechanical load. This can be used to either develop tissue engineered implants or to better study the effect of mechanical load on tissue healing by simulating and predicting in vivo processes [268]. It is quite challenging to achieve and faithfully reproduce complex in vivo load, as the motion pattern can vary greatly even within the same joint [268]. It is necessary to consider that during load the cartilage deformation and shear due to the rotation of the femur to the tibia is different in the different sites of the tibiofemoral joint of the knee [269,270]. Nevertheless, even rudimentary mechanical stimulation is more desirable than none.

Notably, the use of bioreactor systems and loading devices to reproduce the knee environment is emerging more and more in recent years mainly because through the support of the bioreactors. Using bioreactors, it is possible to apply a range of different forces, both alone or combined, recapitulating the complex motion found in in vivo situation by reproducing the so-called complex multi-axial load. As such, hydrostatic pressure, compression, shear, tension or a combination of the mentioned forces in a static and dynamic manner can be attempted [261,268]. 

The spinner flask was one of the easiest fluid-based bioreactor attempts for the development of cartilage-like constructs. This type of bioreactor is not ideal for the development of a three-layer cartilage constructs due to the formation of zones of fibrous tissue at the border of the construct [271,272,273]. Laminar flow bioreactors have been more successful compared with spinner flasks as it was possible to produce tissues with bulk mechanical properties and GAG content that better resembles the native tissue due to a higher GAG level into the middle of the tissue and lower GAG content in peripheral zones [274,275]. Flow perfusion bioreactors were developed to ensure the delivery of fresh medium and to remove waste products by effectively pumping fluid through porous scaffolds. In addition, the movement of the fluid has a potentially positive effect due to the application of a fluid shear force upon the cells embedded in the scaffold. It was shown that limited fluid shear is particularly beneficial for bone formation by promoting osteoblast differentiation, proliferation, upregulation of angiogenic and osteogenic factors, and mineralized matrix production [276]. Dual flow bioreactor systems have been developed to allow for separate nutrition of the bone and cartilage [277].

Physical stimuli can also be transmitted through the fluid medium in bioreactors producing hydrostatic pressure gradients or fluid flow across or through a construct [278]. Therefore, also bioreactors based on hydrostatic pressure have successfully affected the behavior of chondrocytes, depending on the zonal organization, with an increased GAG content in those chondrocytes residing in the middle zone of the construct [279,280]. 

Mechanical stimuli may also be applied directly to constructs in the form of static or dynamic tension, compression, or shear [281]. It is also necessary to consider that mechanical loading due to normal functional activities can be destructive to the regenerative process either by causing outright failure or shunting repair down to a deleterious (e.g., fibrotic) pathway. Conversely, mechanical signals can also have a stimulatory effect on tissue regeneration and are believed to be necessary to achieve full restoration of function [282]. 

Also, shear is an important stimulus. Specific shear motion parameters to stimulate collagen proteins and proteoglycan synthesis in bovine cartilage explants were identified by Jin et al. [283]. 

A good balance among direct compression, rolling movement, shear motion, hydrostatic pressure and tensile forces may be useful to recapitulate the complex motion affecting articular cartilage [262,270]. Different types of bioreactors have been developed to better reproduce the nature of the joint motions and particularly the continuous passive motion (CPM) which is part of patient rehabilitation regimens after a variety of orthopedic surgical procedures because enhance the joint healing process. 

One of the first attempt to potentially provide an in vitro system for the evaluation of clinical strategies of continuous passive motion (CPM) therapy to promote cartilage remodeling was done by Sah et al. The biosynthetic response of calf articular cartilage explants to dynamic compression over a wide range of amplitudes, waveforms, and frequencies was analyzed and dynamic compression was identified as important mechanical stimulus to modulate chondrocyte biosynthesis [284]. The chondrocyte behavior under static and dynamic compression was also studied by Buschmann et al. They found that chondrocytes seeded in agarose gel exhibit a biosynthetic response to compression similar to explanted cartilage. This response was significantly affected by the presence or absence of matrix, suggesting the importance of cell-matrix interactions rather than matrix-independent cell deformation [285]. 

By using a whole-joint bioreactor properly designed to mimic CPM with bovine stifle joints in vitro, it was observed that CPM has a direct effect on the regulation of articular cartilage due to the stimulation of the chondrocyte proteoglycan 4 (PRG4) metabolism [286].

Due to the complex multiaxial load necessary to mimic as closely as possible the forces present in the in vivo environment, a more tribological approach was also suggested. Tribology is defined as the science and technology of interacting surfaces in relative motion [287]. 

In line with this approach, effort was made to translate the interaction of the articular cartilage surfaces that occur in natural knee joint into a bioreactor concept for articular cartilage engineering. As such, a joint-simulating bioreactor, properly designed to mimic more closely joint kinematics and consisting of a rotating scaffold and/or cartilage pin onto a rotating ball, was developed [288]. By oscillating pin and ball and by simultaneously applying dynamic compression, is possible to better reproduce the in vivo motion. By comparing the free-swelling control and/or simply compression-loaded samples with those samples exposed to complex multiaxial load, an increased expression of cartilage matrix genes was observed in the latter group. This loading device enabled a better study of the initial pathways of mechanotransduction by chondrocytes, with particular attention to the shear forces to achieve more successful cartilage tissue engineering [288]. These studies suggest that the application of mechanical forces of engineered joints influence the tribological properties of the synovial interfaces, which in turn would affect the local mechanobiological environment of the cells within articulated tissues. Mechanical stimulation of osteochondral explants is also possible [289].

**Figure 7:**
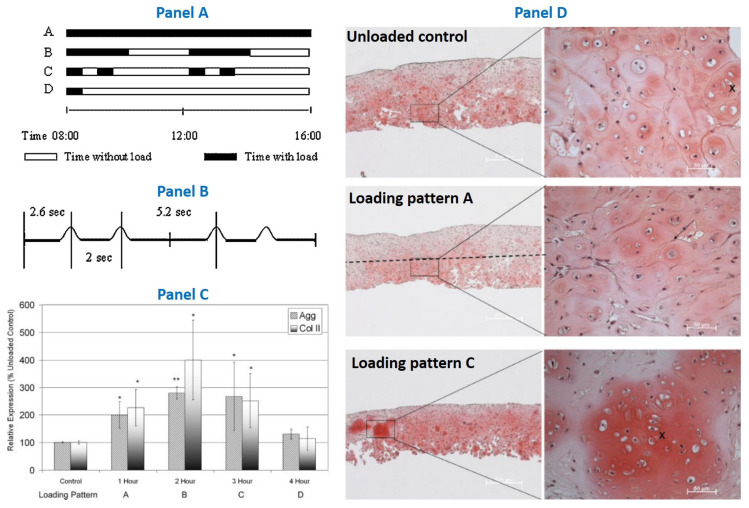
Dynamic load applied for 2 h each morning and afternoon (loading pattern B) enhance matrix synthesis and incorporation while dynamic load applied for 8 h a day (loading pattern A) appear to be detrimental for extra cellular matrix deposition. This finding may be of significant clinical relevance when planning rehabilitation of patients recovering from cartilage repair treatments which involve cell transfer or immature tissue. Schematic illustration of the loading patterns applied (**panel A**): cyclical loading for 8 h a day (loading pattern A); cyclical loading for 2 h in the morning and afternoon (loading pattern B); two times 30 min in the morning and afternoon (loading pattern C); cyclical loading for 30 min in the morning (loading pattern D). Timing of load within one loading cycle (**panel B**). Gene expression levels (**panel C**) of type II collagen (Col II) and aggrecan (Agg) (* *p* < 0.05, ** *p* < 0.01). Safranin O staining (**panel D**) after 4 days of unloaded control, cyclical loading for 8 h a day (loading pattern A) and two times 30 min in the morning and afternoon (loading pattern C) Arrows highlight the PCM border developed when the cells were grown in alginate beads. Loading pattern C lead to more fused matrix (x) showing more integration between the individual chondrons. Images modified with permission from M.J. Stoddart et al., 2006 [290].

Another bioreactor that enables the application of shear associated with compression simultaneously to up to 20 constructs with four different types of loading patterns, was used to subject the de novo cartilage-like tissue construct to mechanical load [290] (Figure 7). It was observed an increased gene expression of type II collagen and Aggrecan associated with an increased GAG level when the load applied was intermittent. This study developed a mechanical stimulation protocol that enhances matrix deposition in de novo cartilage constructs and improves the properties of the engineered tissue prior to implantation. The important finding of this study was essentially associated to the timing program of the load applied. Notably the study suggests that introducing pauses between load cycles is beneficial for the construct development and leads to a reproducible increase in GAG/DNA. In contrast, constant cyclical load, lead to a decrease in the final GAG content. This finding may be of significant clinical relevance for two main reasons: it may be useful to improve the rehabilitation protocol of patients recovering from cartilage injury and may be helpful to increase the clinical effectiveness of the de novo engineered cartilage-like constructs for implantation purposes [290].

It is well known that biomechanical forces are involved in bone remodeling and repair but the underlying mechanisms by which a physical force is translated to the corresponding intracellular signal is not yet completely understood. Mechanotransduction is a critical feature to be considered in the design of a proper bioreactor system [291]. Mechanosensitivity is an essential property of all organisms from bacteria to humans and the physical forces regulate a large array of physiological as well as pathological processes by altering protein conformation, folding, phosphorylation or channel structures to generate different cascade signals [292,293].

In the engineering of a partial or whole joint as well as in the design, development, fabrication and testing of a bioreactor system, the role of shape, loading and motion of synovial joint mechanobiology is crucial [281]. 

**Figure 8:**
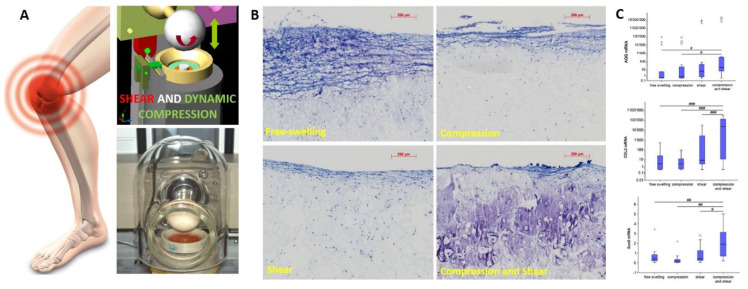
Shear and dynamic compression are critical component to achieve successful mechanical loading induced-chondrogenesis in human mesenchymal stem cells. This finding can be useful to develop better rehabilitation protocols for MSCs-mediated cartilage repair. Joint-simulation bioreactor capable to apply multiaxial loading miming knee joint motions (**A**). Shear in combination with compression is crucial for the enhanced deposition of proteoglycan-rich extra-cellular matrix as showed in toluidine blue staining (**B**) and for the upregulation of the cartilage gene expression markers Sox9, COL2 and Aggrecan. (**C**) Relative mRNA expression of human mesenchymal stem cells after 21 days, * and ° depict outlier samples. # *p* < 0.05, ## *p* < 0.01, ### *p* < 0.001. Images modified from with permission Schatti et al., 2011 [295].

Human stem cells have shown a different responsiveness to mechanical loading compared with chondrocytes [294], namely that compression alone is not sufficient to induce chondrogenesis of MSCs [295] (Figure 8).

It was observed that bioreactors that incorporate dynamic compression at physiological strain levels enhanced chondrocyte matrix elaboration in cell-seeded agarose scaffolds and produced a more functional engineered tissue construct than in free swelling controls [253]. Later, it was observed that bovine MSCs in agarose required a period of TGF-β induced chondrogenesis prior to the application of load [296] while an early application of cyclic compression on porcine MSCs also was detrimental to chondrogenesis [297]. Thus, the response of MSCs to load was seen to be different to that observed when using chondrocytes. A chondrogenic induction was demonstrated with human MSCs and multiaxial load in the absence of TGF-β [298,299]. The conflicting outcome was shown to be as a result of the absence of shear in the uniaxial compression studies, and in agreement with the other studies, compression alone did not lead to chondrogenic induction [295]. This has been shown to be due to a mechanical induction and activation of endogenous latent TGF-β [300,301]. Bioreactors that incorporate compression and shear motion have also been extremely successful in investigating the response of chondrocytes to various loading regimes [288,290]. It was observed a markedly different tissue depending to the motions applied [302]. Notably, sliding-type biomechanical stimuli may favor regeneration and maintenance of functional and operative articular surfaces and support the development of mechanically competent engineered cartilage. This has implications for both in-vitro tissue engineering as well as in vivo physical regenerative therapy regimes.

Finally, another parameter to be considered is the importance of the synovial fluid present in the cavities of synovial joints [303]. Synovial fluid is a viscous, non-Newtonian fluid and its principal role to reduce friction between the articular cartilage of synovial joints during movement. The presence of a synovial fluid mimicking media, which can better simulate the rheological and biological features of synovial fluid, would be of great advantage to recapitulate the in vivo environment by modifying the chondrogenic response to multiaxial load.

## 10. Conclusions and Future Perspectives

The most advanced ex vivo models include the co-culture of at least two different cell types combined with proper tissue engineering strategies and loading motions aiming to reproducing the complex structure and function of the native tissue by using biomimetic scaffolds and suitable biological cues. These models represent reliable systems to reduce the gap existing between the complexity of the in vivo environment and the simplicity of in vitro condition thereby decreasing the needs of animal studies. As such, the ex vivo systems are useful tools to investigate, in a more controlled environment, the complexity of the in vivo physiological and pathological processes and in so doing, they allow to better prevent in vitro artefacts and to achieve more truthful results if compared with previous simpler models. 

**Figure 9:**
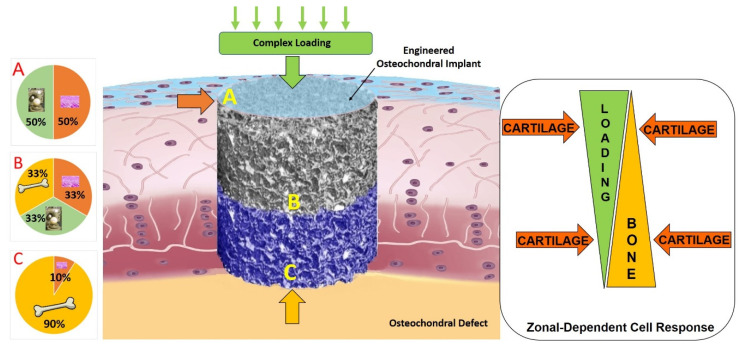
Zonal dependent cell response of de novo cell-based engineered osteochondral implant within and osteochondral defect. The different location of the cells inside the osteochondral implant might affect cell behavior, differentiation, tissue maturation and integration due to the different signaling coming from the native surrounding tissue and from the loading and due to the intensity of the signal determined by the distance between the source of the stimulus and cells. Cells reside in position A will be equally affected by loading motions and cartilage but minimally by the bone; cells reside in position B will be equally affected by the loading motions, cartilage and bone signaling; cells reside in position C will be affected mainly by the bone, partially by the cartilage and minimally by the loading.

The further development of new ex vivo models approximating an in vivo environment, is a promising approach to improve our knowledge of the biological mechanisms underlying cartilage regeneration process. Future studies should aim to better elucidate the crosstalk mechanisms between the different cell types involved in osteochondral repair and should consider that several cytokines secreted by bone cells can lead to chondrocyte differentiation [304]. Understanding the dialogue between cartilage and underlying bone might be the key to shed light on the molecular signaling pathways of physio-pathological conditions and may help to restore the healthy situation. The dialogue between the different cell types might also be affected and regulated by the location of the cells within the tissue and the distance between the source of the stimulus. Thus, a specific stimulus might direct cell behavior as a zonal-dependent cell response (Figure 9).

Indeed, it is also noteworthy to highlight that biomechanical factors profoundly influence the processes of tissue growth, development, maintenance, degeneration, and repair. Therefore, the ability to apply joint kinematic motion through the appropriate bioreactors allows for a more physiological system to study in-vitro or ex-vivo cartilage regeneration mechanisms, to create living tissue replacements and to test new potential cartilage repair therapies.

## Data Availability

Data sharing not applicable. No new data were created or analyzed in this study. Data sharing is not applicable to this article.
